# LXR agonism for CNS diseases: promises and challenges

**DOI:** 10.1186/s12974-024-03056-0

**Published:** 2024-04-16

**Authors:** Ruiyi Zhang, Emily Wuerch, V. Wee Yong, Mengzhou Xue

**Affiliations:** 1 Department of Cerebrovascular Diseases, The Second Affiliated Hospital of Zhengzhou University, Zhengzhou, Henan China; 2 Hotchkiss Brain Institute and Department of Clinical Neurosciences, University of Calgary, Calgary, AB Canada

**Keywords:** Liver X receptor, Neurological diseases, Neuroinflammation, Cholesterol metabolism, Tissue regeneration, Clinical translation

## Abstract

The unfavorable prognosis of many neurological conditions could be attributed to limited tissue regeneration in central nervous system (CNS) and overwhelming inflammation, while liver X receptor (LXR) may regulate both processes due to its pivotal role in cholesterol metabolism and inflammatory response, and thus receives increasing attentions from neuroscientists and clinicians. Here, we summarize the signal transduction of LXR pathway, discuss the therapeutic potentials of LXR agonists based on preclinical data using different disease models, and analyze the dilemma and possible resolutions for clinical translation to encourage further investigations of LXR related therapies in CNS disorders.

**Graphical Abstract:**

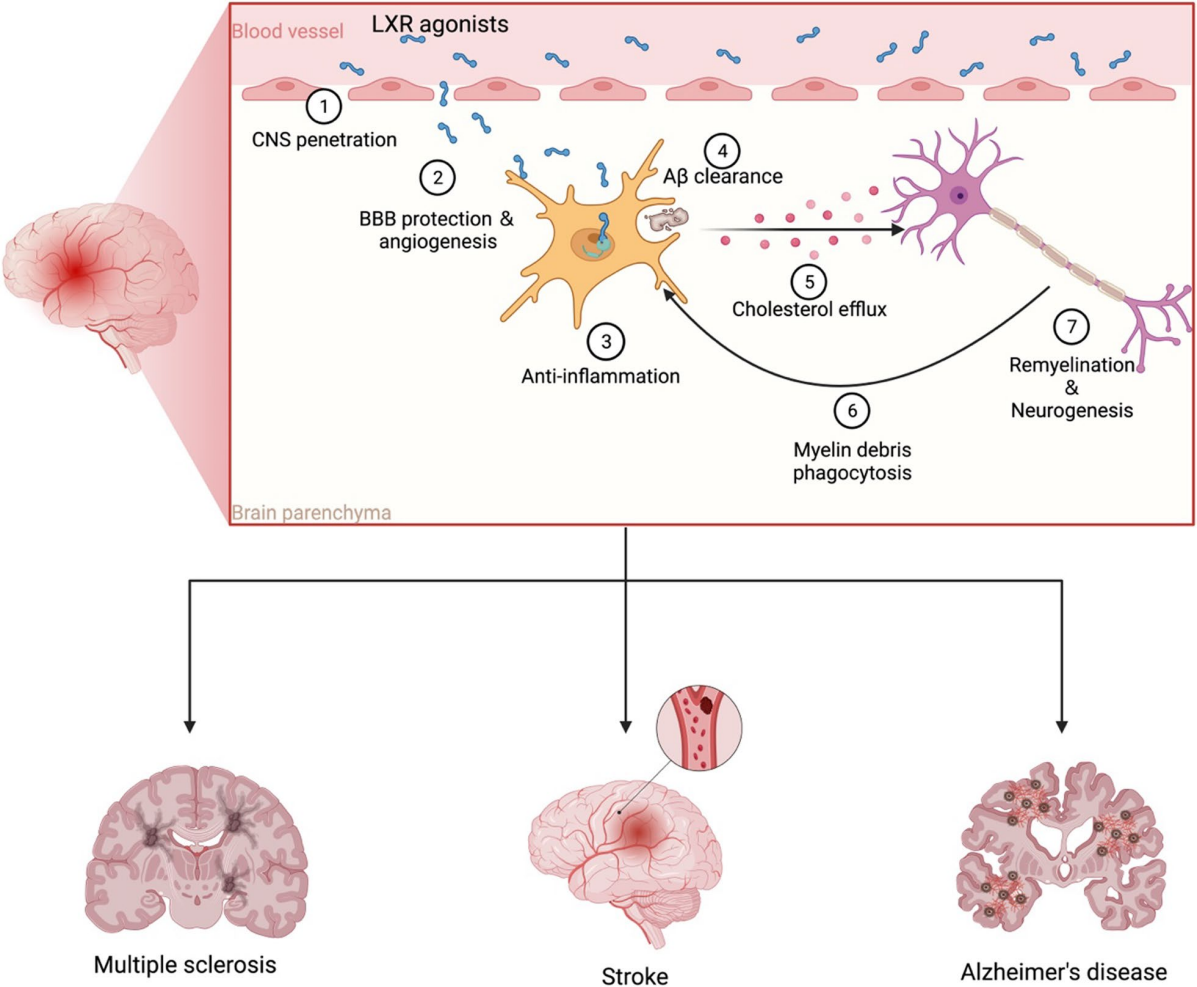

## Background

A novel type of orphan nuclear receptors was discovered in 1994 [1] and received its name of LXR (see Glossary) the next year. There are two isotypes of LXR, LXRα and LXRβ, which share up to 77% sequence homology but have distinct distribution [2]. LXRα is highly expressed in tissues critical for lipid metabolism including liver, intestine and adipose tissue, and in cell types such as macrophages [3]. LXRβ is ubiquitously expressed but is particularly prominent in the liver and brain [4].

Research on LXRs started from, and continues, in the realm of endocrinology and cardiovascular medicine. However, LXRs have attracted increasing interests from the neurosciences due to its involvement, and thus potential clinical translation and intervention, in many refractory CNS diseases. Here we review the biology of LXRs, summarize the preclinical results of LXR in models of neurological disorders, and consider its potential as a therapeutic target in CNS diseases. We focus on the functions and underlying mechanisms of LXRs, and hope that our discussions spur further exploration into the translation of LXR targets in neurology.

## Overview of signal transduction of LXRs

**Fig. 1:**
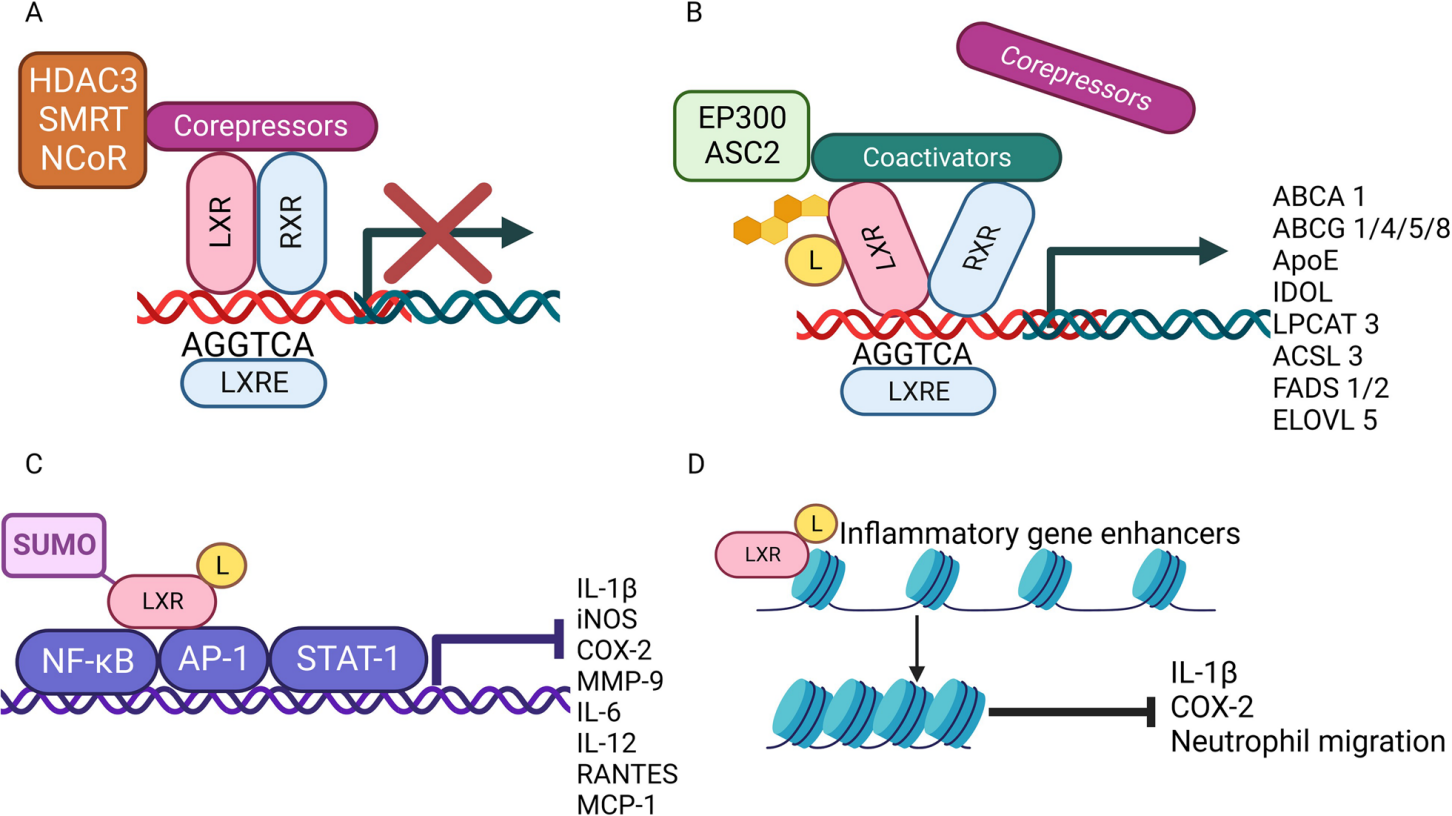
Signal transduction of liver X receptors. **A** Inactive state: LXR-RXR complex binds to corepressors when ligands are absent, and downstream gene transcription is inhibited. **B** Activation: endogenous or synthetic ligands (L) trigger conformational change of LXR-RXR complex, leading to the release of corepressors and recruitment of coactivators and downstream gene transcription. **C** Trans-repression: SUMOylated ligand-binding LXR monomer interacts with NFκB, AP-1 and STAT1, inhibiting pro-inflammatory gene transcription. **D** Cis-repression: ligand-binding LXR monomer conjugates to pro-inflammatory gene enhancers, mediating chromatin closing and inhibiting pro-inflammatory gene transcription. *ABCA 1* ATP binding cassette subfamily A member 1, *ABCG 1/4/5/8* ATP binding cassette subfamily G member 1/4/5/8, *ACSL3* Acyl-CoA synthetase long chain family member 3, *AP-1* activator protein 1, *ASC2* activating signal cointegrator 2, *ELOVL 5* elongation of very long chain fatty acids protein 5, *FADS 1/2* fatty acid desaturase 1 and 2, *HDAC3* histone deacetylase 3, *IDOL* inducible degrader of low-density lipoprotein receptor, *iNOS* inducible nitric oxide synthase, *LPCAT3* lysophosphatidylcholine acyltransferase 3, *LXRE* LXR response element, *MCP-1* Monocyte chemoattractant protein-1, *MMP-9* matrix metalloproteinase-9, *NCoR* nuclear receptor corepressor, *NF-κB* nuclear factor kappa B, *RANTES* regulated upon activation, normal T cell expressed and presumably secreted, *SMRT* silencing mediator for retinoid and thyroid receptors, *STAT1* signal transducer and activator of transcription 1

Natural ligands of LXRs include cholesterol derivatives such as 22(R)-hydroxycholesterol, 24(S),25-epoxycholesterol, 24(S)-hydroxycholesterol, 25-hydroxycholesterol, 27-hydroxycholesterol and cholic acid. LXRs form heterodimers with retinoid X receptor (RXR), and the dimer binds to a specific DNA region containing repetitive AGGTCA sequence named as LXR response element [5]. When ligands are not present, the LXR-RXR complex binds to corepressors such as histone deacetylase 3, silencing mediator for retinoid and thyroid receptors or nuclear receptor corepressor and remain inactivated [5] (Fig. 1). In this inactive state, downstream genes including apolipoprotein E (ApoE), ATP-binding cassette transporter sub-family A and G (ABCA, ABCG), or inducible degrader of LDLR (IDOL) remain inhibited [5]. With the binding of endogenous or synthetic ligands such as GW3965 and T0901317, the conformational change of LXR-RXR heterodimers results in the release of corepressors and the recruitment of coactivators such as histone acetyltransferase p300 and activating signal co-integrator 2 [6]. These sequentially induce the expression of target genes to facilitate cholesterol and lipid metabolism [7]. Additionally, ligand-bound LXRs can be SUMOylated to interact with NFkB, AP-1 and STAT1, leading to the trans-repression of their downstream proinflammatory genes [8, 9]. Moreover, analyses by transposase-accessible chromatin sequencing and chromatin immunoprecipitation sequencing show that activated LXRs bind inflammatory gene enhancers to induce chromatin closing, resulting in cis-repression of these genes in LPS-treated bone marrow derived macrophages [10].

## LXR in lipid homeostasis

### Cholesterol clearance

The brain, comprising only 2% of body weight, contains around 20% of the total unesterified cholesterol in the human body [11]. 70–80% of the brain cholesterol participates in the formation and maintenance of myelin while the rest are crucial components of cellular membranes [11]. Due to the existence of the blood brain barrier (BBB), circulating lipoproteins in the periphery can barely enter the CNS so that the de novo synthesis and clearance of brain cholesterol are regulated by a distinct CNS-specific system [11], where LXRs play a pivotal role.

Cholesterol metabolism in the CNS is tightly regulated in homeostasis. Developing neurons depend highly on intracellular cholesterol synthesis to maintain normal growth and survival [12], while mature neurons predominantly harness the cholesterol generated by glial cells, particularly astrocytes through reverse cholesterol transport, to maintain membrane fluidity and cellular functions [13]. A genetic deficiency of 7-dehydrocholesterol reductase, an enzyme required for cholesterol biosynthesis, leads to Smith-Lemli-Opitz Syndrome with interrupted CNS development and function, characterized by growth restriction, microcephaly, intellectual disability, and multiple malformations [14].

**Fig. 2:**
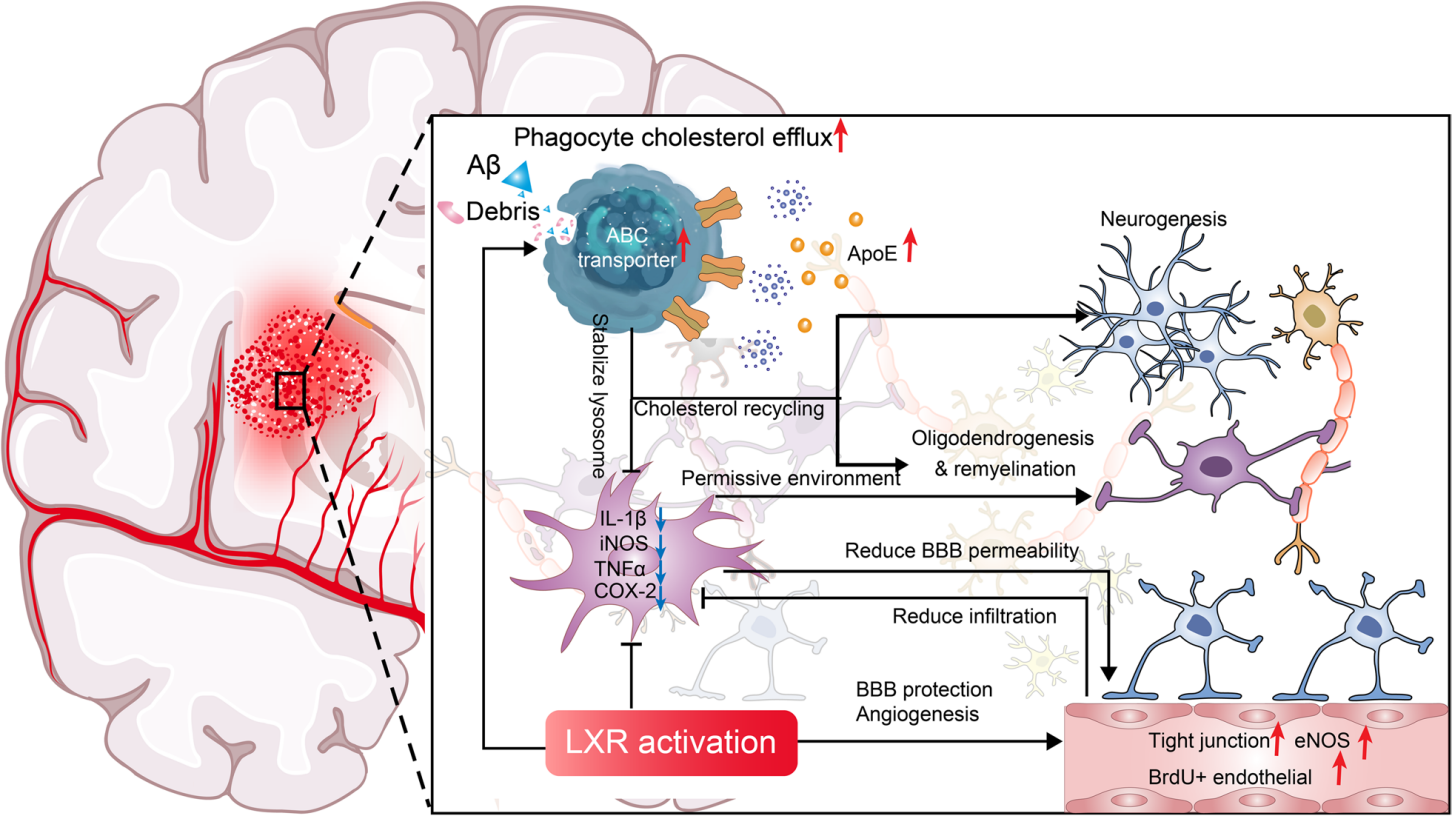
The functions of LXR signaling in CNS pathology. The activation of LXR may inhibit neuroinflammation directly by transcriptional repression and indirectly by cholesterol efflux and following lysosome stabilization, which together provides a more favorable microenvironment for regeneration. The enhanced cholesterol efflux from phagocytes can also provide material for tissue repair by oligodendrocyte precursor cells (OPCs) and neural stem cells. Upregulated ApoE expression and esterification may facilitates beta-amyloid clearance. Enhanced BBB integrity and angiogenesis by LXR agonist were also documented, but the potential mechanisms remain unknown. *ABC transporter* ATP-binding cassette transporter, *ApoE* apolipoprotein E, *Aβ* amyloid beta, *BBB* blood–brain barrier, *BrdU* bromodeoxyuridine, *COX-2* cyclooxygenase-2, *eNOS* endothelial nitric oxide synthase, *IL-1β* interleukin 1 beta, *iNOS* inducible nitric oxide synthase, *TNFα* tumor necrosis factor α

Conversely, accumulation of excessive cholesterol in neurons and glia is associated with multiple neurological malfunctions, such as Niemann-Pick type C disease, Cerebrotendinous xanthomatosis and demyelinating conditions. Here, the feedback response of LXRs is crucial in lowering the elevated intracellular cholesterol level by both reducing uptake and promoting efflux from a cell [9, 15]. When binding to oxysterols or synthetic ligands, the conformational change of LXR-RXR complex induces a series of gene transcription related to lipid regulation. To hinder the intake of cholesterol, LXRs directly targets the promoter of IDOL, which mediates endocytosis and degradation of low density lipoprotein receptor as a type of E3 ubiquitin ligase resulting in reduced cellular uptake of low density lipoprotein [16]. As for the reverse cholesterol transport, LXR activation promotes the generation of both channel and vehicle for this process (Fig. 2). As cholesterol efflux regulatory proteins, several members of ABC transporters including ABCA1, ABCG1/4/5/8 are transcriptionally regulated by LXRs [15]; these membrane transporters open the gate and translocate sterols outside of cells through conformational change after substrates bind and ATP drives the process [17]. Next, the combination with ApoE, the dominant apolipoprotein in brain controlled by LXRs, is necessary for the removal from or recycling in the CNS of the effluxed cholesterol [18]. Based on this ability to regulate multiple genes, LXRs localize in the nexus of cholesterol trafficking in the CNS, and its dysregulation thus leads to various neurodegenerative pathologies.

In contrast to the Smith-Lemli-Opitz Syndrome caused by cholesterol deficiency, Niemann-Pick type C disease results from inherited *NPC1* or *NPC2* gene mutations that produce intracellular lysosomal cholesterol overload particularly in the CNS [19]. T0901317, a synthetic LXR agonist, increases ABCA1 expression and subsequent cholesterol efflux in human NPC1 deficient fibroblasts [20]. In mice modeling Niemann-Pick type C disease, treatment with the same agonist elevated brain ABCA1 and ABCG1 expression, cholesterol offloading and lifespan, in addition to decreased cerebellar inflammation [21].

Since ApoE, particularly lipidated ApoE, mediates the clearance of β-amyloid (Aβ) [22], the canonical driver of Alzheimer’s disease (AD), LXRs have received significant attention in this neurological condition. In a recent study, T0901317 alleviates β-amyloid induced neurotoxicity in human neural stem cell scaffolds and AD mice, which is hindered by the injection of GSK2033, a LXR antagonist [23]. Treatment with LXR agonists in different transgenic models of AD improves behavior performance and/or ameliorates pathology [15, 24]; however, not every study finds the reduction of Aβ or plaques [25], and some even report increased soluble β-amyloid when treated with low dose GW3965 [26]. This phenomenon may be attributed to the multigenic regulation of LXR and the complexity of cellular interaction in CNS. For example, the increasing low-density lipoprotein receptor (LDLR) induced by IDOL deletion or pharmaceutical inhibition seems to promote ApoE mediated Aβ removal and slows functional deterioration [27]. In contrast, the genetic silencing of ABCA1 worsens Aβ deposition [26]. Unfortunately, both IDOL and ABCA1 are upregulated by LXR activation, which may partially explain such contradictory observations in AD models. Moreover, even while ApoE is crucial for Aβ clearance, the ε4 allele (ApoE4) is considered a risk factor for AD. ApoE4 appears not only less protective than other genotypes but toxic as well [28], which brings more uncertainty to LXR agonism in AD treatment. The RXR agonist bexarotene shows an ApoE dependent manner of rapid Aβ reduction and cognitive enhancement in APP/PS1 mice, while reversal of memory deficits but not ameliorated Aβ deposition is observed in mice expressing human ApoE3 or E4 isoform [29]. In the most recent study using P301S tau mouse model of AD, it has been observed that ApoE4 markedly exacerbates lipid accumulation within glial cells, alongside disturbances in cholesterol metabolism and lysosomal functioning [30]. However, interventions aimed at increasing lipid efflux in glial cells, particularly through the activation of LXR by GW3965 or overexpression of Abca1, have demonstrated significant mitigation of tau pathology, neuroinflammation and neurodegenerative processes in the P301S/ApoE4 mouse model [30]. Despite ongoing debates, these results imply that LXR agonism could play a multifaceted role in mitigating Alzheimer's disease pathology—both by augmenting amyloid-beta clearance and by regulating neuroinflammation associated with the disease through the reduction of lipid accumulation in glial cells. This multifunctionality underscores the continued promise of LXR agonists as a research focus in Alzheimer's disease therapeutics.

### Regulation of membrane components

Phospholipids are the basic components of membranes and myelin. They are sensitive to oxidative stress injury due to the *sn-2* polyunsaturated fatty acid side chain being easily oxidized, followed by generation of oxidized phospholipids as end-point product in various CNS pathologies [31]. Oxidized phospholipids are not only markers but also mediators of oxidative injury by exacerbating inflammation and cell death [31], possibly due to active carbonyl or hydroxyl generated by previous peroxidation, while antibody neutralization was observed to ameliorate pathology in non-CNS disease models [32, 33]. Recently, we found that oxidized phosphatidylcholines are neurotoxic in vitro and induce axonal degradation and neuroinflammation in vivo, and antibody neutralization limits neurodegeneration and apoptosis [34].

LXRs modulate the enzymes required for polyunsaturated fatty acid synthesis including ACSL3, which adds the acyl-CoA to fatty acids; FADS1/2, which desaturates the substrate; and ELOVL5, which elongates the PUFA chain [35]. More importantly, as a direct activator of lysophosphatidylcholine acyltransferase 3 that catalyzes polyunsaturated fatty acids to *sn-2* site of lysophospholipids, LXR activation increases the abundance of the polyunsaturated fatty acid-incorporated phopsholipids in membrane structures and ameliorates oxidative stress [35]. Even though there is currently no data detailing phospholipid turnover and LXRs in neurological disorders, it is plausible to hypothesize that LXR may provide neuroprotection through membrane phospholipid rejuvenation. We have observed alleviated neurodegeneration and apoptosis by LXR agonist treatment in lesions induced by oxidized phosphatidylcholines in the spinal cord [36].

## LXR in neuroinflammation

Ligand-bound LXRs can be SUMOylated to exert trans-repression targeting NF-κB and AP-1 [9], which are transcriptional activators of several proinflammatory genes implicated in neuropathological processes [37]. Interestingly, this inflammation-inhibitory property seems independent of RXR, through the SUMOylated LXR acting as a monomer to consolidate the connection between nuclear receptor corepressor and NF-κB or AP-1 [9] (Fig. 1C). In addition, activated LXRs are reported to bind to enhancers of proinflammatory genes to induce chromatin closing, resulting in cis-repression [10] (Fig. 1D). Synthetic or natural LXR agonists reduce expression of proinflammatory molecules including IL-1β, iNOS, TNFα, MCP-1, COX-2 and nitric oxide production in LPS-treated microglia, astrocytes or leucocytes in culture, which is likely attributed to the inhibition of NFκB and AP-1, while LXR inhibition enhances inflammation [38–40].

LXR activation has shown benefits in models of several neurological diseases through coping with excessive or dysregulated neuroinflammation. Besides Aβ deposition and p-tau indued neurofibrillary tangles, uncontrolled inflammation may worsen Alzheimer’s disease. LXR activation attenuates Aβ triggered microglial inflammatory responses in vitro, and reduces iNOS, COX-2 and nuclear NFκB level in APP/PS1 transgenic mice [41, 42]. Similar effects were observed in the MPTP model of Parkinson’s disease, where the level of iNOS and COX-2 level was attenuated with treatment of T0901317 [43].

Overwhelming inflammation-induced secondary brain injury contributes to poor prognosis of stroke even with thrombectomy or hematoma removal [44]. In a rat model of global cerebral ischemia induced by carotid artery clipping, one dose of 20 mg/kg GW3965 reduced NFκB p65 translocation and COX-2 levels 12 h after injury; this was followed by improved neuronal survival and behavioral performance on day 7 [45]. In a permanent middle cerebral artery occlusion (MCAO) model of ischemic stroke, GW3965 or T0901317 treatment resulted in decreased NFκB transcriptional activity and downstream inflammatory gene transcription including IL-6, IL-12, RANTES and MCP-1; reduced expression of IL-1β, iNOS, COX-2 and MMP-9 was also observed at 24 or 48 h after injury [46, 47]. As for hemorrhagic stroke, T0901317 alleviated neuroinflammation manifested by reduced IL-6, iNOS, COX-2 and chemokines, and this correlated with reduced neutrophil infiltration, lesion volume and neurodegeneration 4 days after collagenase induced intracerebral hemorrhage (ICH) [48]. In our recent study, daily administration of 10 mg/kg GW3965 for 7 days after collagenase induced ICH inhibited the expression of IL-1β and iNOS at perihematomal region. More importantly, GW3965 promoted the phenotype shifting of perihematomal microglia/macrophages from proinflammatory (IL-1β^+^Iba1^+^) to regulatory phenotype (Arg1^+^/CD206^+^ CD68^+^) at day 7 post-ICH; this phenotype change was associated with improved histological and functional recovery because conditional depletion of microglia/macrophages abrogated the therapeutic effects of LXR agonism [49].

Phagocytes are required to clear cholesterol-enriched components including cellular and myelin debris after CNS injury. However, the overloaded cholesterol could form crystals that destabilize lysosome, resulting in leakage of cathepsin B, induction of inflammation through activation of NLRP3 inflammasome activity, cleavage of caspase-1 and following IL-1β releasing [50]. In LPS-primed human and mouse peripheral blood mononuclear cells (PBMCs), intracellular cholesterol crystals increased the expression of IL-1β in a concentration-dependent manner [51]. Moreover, the downregulated proinflammatory genes and microgliosis in experimental intracerebral hemorrhage were associated with reduced accumulation of crystals and lipid droplets [49]. Even though the direct proof is lacking to conclude that excessive cholesterol from myelin degradation and cellular debris worsen neuroinflammation in different CNS pathologies, it is plausible to hypothesize that microglia and macrophages may act in this manner. If so, LXR activation would alleviate inflammation by cholesterol clearance in addition to direct anti-inflammatory activity.

## LXR and the blood–brain barrier

An intact blood–brain barrier (BBB) is crucial for homeostasis and functioning of the CNS. Unfortunately, this barrier is susceptible to various kinds of injury leading to leakage of toxic substances and the infiltration of inflammatory cells into the CNS parenchyma with resultant pathology. LXRs are expressed in brain capillary endothelial cells that are essential components of the BBB. LXR activation by natural agonist oxysterol or RXR activation by bexarotene reduced the permeability of a blood brain barrier model of endothelial cells co-cultured with glia; this may be attributed to increased expression of ABCB1 [52], a member of ABC transporters located in endothelial cells to limit the entrance of potential toxins into the brain [53]. On the contrary, LXRα knockdown impaired the integrity of endothelial barrier and reduced tight junction protein accompanied by increased vascular cell adhesion molecule-1 and inflammatory infiltration both in vitro and in vivo of the experimental autoimmune encephalomyelitis (EAE) model of multiple sclerosis [54]. In a stroke model of 30-min transient MCAO, both GW3965 and T0901317 improved the expression and phosphorylation of occludin, and its assembly with ZO-1, which are components of tight junction belonging to endothelial barrier of the BBB; and this protection may result from the reduction of calpain-1/2 and MMP-2/9 [55]. Moreover, the authors also reported the increase of capillary endothelial ABCB1 and alleviated brain edema with treatment [55].

## LXR promotes CNS tissue regeneration

The CNS has some degree of self-regenerative ability following a variety of pathologies [56, 57]. LXRs and their related pathways have been shown to affect tissue repair and functional recovery in multiple preclinical disease models.

### Neurogenesis

LXR stimulation facilitates neurogenesis of midbrain and dentate gyrus during neural development in zebrafish and rodents [58, 59]. In mice, LXRs regulate the balance between neurons and glia during early midbrain development [60]. LXR-deleted mice show decreased neurogenesis in ventral midbrain during development and lower number of dopaminergic neurons at birth, accompanied with an accumulation of neural progenitors and radial glia cells [60], while the natural ligand oxysterol promotes neurogenesis of both human and mouse embryonic stem cells [59].

In the adult rodent brain, LXR activation is observed to promote post-injury neurogenesis in different disease models. In the MCAO model of ischemic stroke, 30 mg/kg LXR agonist T0901317 treatment for 14 days increased the number of surviving axons and synaptophysin level [61]. Following oxygen glucose deprivation in primary cortical neuronal culture, T0901317 improved neurite outgrowth [61]. The oral gavage of 10 mg/kg GW3965 increased white matter (axons and myelin sheath) density and promoted functional recovery after distal MCAO, while this effect was negated by ABCA1 knockout [62]; this suggests that the protection by LXR activation is partly induced by enhanced cholesterol efflux. In a model of bilateral carotid artery stenosis, 10 mg/kg intraperitoneal injection of GW3965 promoted proliferation of neural stem cells in the dentate gyrus during chronic cerebral hypoperfusion, and improved performance in Morris water maze test [63]. In collagenase induced experimental ICH, 7-day treatment with 10 mg/kg GW3965 increased the density of SOX2^+^Ki67^+^ neural stem cells and SOX2^+^DCX^+^ neuronal progenitor cells in subventricular zones [49]. In a triple-transgenic mouse model of Alzheimer’s disease, 50 mg/kg GW3965 affected gene methylation related to neurogenesis and synaptogenesis including that of Sox-3 and syn1, which may contribute to the improvement in cognitive function [64].

### Angiogenesis

The reconstruction of regional vasculature is crucial for reperfusion and functional recovery after ischemic stroke. MCAO mice showed increased BrdU^+^ proliferating vWF^+^ endothelial cells and αSMA^+^ small arteries as well as elevated occludin level by 14-day of 10 mg/kg GW3965 treatment [65]. 30 mg/kg T0901317 treatment for the same period upregulated endothelial nitric oxide synthase (eNOS) level in addition to enhanced angiogenesis, which was abrogated by eNOS knockout [66].

### Oligodendrogenesis and remyelination

Cholesterol-enriched myelin sheaths formed by oligodendrocytes are essential for insulation and signal transduction of axons in the CNS. De- and remyelination are in dynamic balance during normal lifespan [67], where LXRs are indispensable. LXRs are pivotal for oligodendrocyte precursor cell (OPC) differentiation, proliferation and maturation [68]. LXR knockout mice have less PDGFRα^+^olig2^+^ OPCs during development and they have lower level of axonal myelination in adulthood [69].

In various pathological conditions, limited remyelination fails to fully regenerate lost myelin due to several reasons including a repair-inhibitory lesion microenvironment [70]. The loss of myelin increases the vulnerability of axons and may result in neuronal demise. The activation of LXRs has been observed to support remyelinating processes in preclinical studies of different disease models. In the EAE model, LXR deleted mice had more severe pathology than control animals [71]. In organotypic cerebellar cultures demyelinated by lysolecithin, both T0901317 and 25-hydroxycholesterol induced activation of LXRs and increased the expression of mature oligodendrocyte markers including myelin basic protein, and the number of myelinated axons [72]. In experimental ICH, GW3965 treatment increased the density of OPCs and mature oligodendrocytes at perihematomal regions and improved white matter integrity [49], which was abolished by microglia/macrophage conditional depletion [49]. The underlying mechanisms may be related to alleviated inflammatory responses and enhanced reuse of cholesterol.

CNS demyelination is accompanied by neuroinflammation, particularly of activation of resident microglia and infiltrating macrophages. In lysolecithin-induced corpus callosum demyelination, the phenotype of microglia/macrophages changes from proinflammatory (CD68^+^iNOS^+^ or CD16/32^+^TNFα^+^) to regulatory (CD68^+^Arg1^+^ or CD206^+^IGF1^+^) state corresponding to early remyelination at day 10 [73]. The depletion of CD206^+^ regulatory myeloid cells using mannosylated clodronate liposomes inhibited oligodendrocyte differentiation in vivo, and conditioned medium from regulatory (exposed to IL-10 or IL-13) microglia promoted OL differentiation and maturation in both cell and organotypic cultures [73]. In collagenase induced ICH, GW3965 reduced the expression of proinflammatory markers such as IL-1β and iNOS while promoted Arg1^+^ and CD206^+^ regulatory microglia/macrophages, which was linked to increased oligodendrocyte lineage cells [49]. Moreover, the unresolved neuroinflammation and cytokines including IL-1β may induce continuing cell death and other cascading responses, which could be challenging for the survival and maturation of OPCs, and remyelination [50]. As described above, the trans-repression of LXRs can inhibit excessive neuroinflammation while helping shift microglia/macrophages into regulatory phenotypes, which may also assist remyelinating processes.

During pathological conditions involving demyelination, the CNS may not generate enough cholesterol for remyelination by de novo synthesis. Instead, oligodendrocytes tend to harness the recycled cholesterol from engulfed and degraded myelin, which is processed and excreted by microglia/macrophages [74]. In the study using autopsy MS tissue, LXRα, LXRβ and LXR downstream genes including ABCA1 and APOE, are abundantly expressed in active lesions, especially in foamy phagocytes, suggesting the activation of LXR signaling [75]. As we mentioned above, the upregulation of LXR downstream genes encoding ABC transporters and ApoE triggered by either natural or synthetic agonists could enhance cholesterol efflux, which may contribute to not only the amelioration of inflammation but remyelination by oligodendrocytes [10, 74]. In our recent study, GW3965 reduced cholesterol accumulation in phagocytes after experimental ICH while more white matter tracts were observed by diffusion tensor imaging and fiber tracking, indicating the enhanced cholesterol efflux might assist potential remyelination [49]. The accumulation of unprocessed cholesterol inside phagocytes can form cholesterol crystals after engulfment of myelin debris during demyelination, which may keep microglia/macrophages more proinflammatory due to the destabilization of lysosomes by crystals and following formation of NLRP3 inflammasomes and activation of caspase-1 and IL-1β [50]. Moreover, persistent inflammation-induced enhanced microgliosis may prevent tissue regeneration physically and biologically [10, 50]. In the lysolecithin model of demyelination, impaired cholesterol clearance induced by aging, ApoE knockout or ABCA1/G1 double knockout in phagocytes elevated inflammatory responses and prevented remyelination after injury [76]. In this regard, LXRα knockout mice showed increased lesion volume, cholesterol crystal accumulation and inhibited recovery, while treatment with the synthetic LXR agonist GW3965 promoted remyelination by rescuing inefficient cholesterol efflux and ameliorating inflammatory responses in the aged CNS [76]. In the same model of western diet fed mice, GW3965 reduced the number of myelin and crystal laden Iba1^+^ microglia/macrophages as well as increased fluoromyelin positive areas in the corpus callosum 14 days after lysolecithin injection [77].

Triggering receptor expressed on myeloid cells 2 (TREM2) has been shown essential for the clearance of myelin debris by microglia [78]. TREM2 deficient mice displayed increased lipid accumulation, especially cholesterol ester inside microglia/macrophages and brain compared to wild type mice when coping with cuprizone induced demyelination, while cell type specific lipidomics showed GW3965 alleviated cholesterol ester burden in phagocytes in both TREM2 knockout and wildtype mice [79].

The enhanced clearance of myelin debris and immune modulation can both facilitate the conversion of an inflammatory microenvironment to a more remyelination-permitting stage. In addition, LXR activation provides material to help oligodendrocytes for myelin synthesis directly: in cuprizone, EAE, lysolecithin and MBP-conditionally null mice, CD11b^+^ myeloid cells downregulated the synthesis of cholesterol by lowering 24-dehydrocholesterol reductase expression, which led to higher intracellular desmosterol, an intrinsic ligand to activate LXRs, and this then boosted cholesterol efflux [74]. In spinal cord culture to mimic oligodendrocyte remyelination in vitro, the natural LXR agonist squalene increased the number of MBP positive axons, which is attributed to utilization of more recycled cholesterol from phagocytes for myelin sythesis [74]. Moreover, squalene treatment upregulated the remyelinating process and improved functional recovery in various preclinical demyelinating conditions in vivo [74].

## Clinical translation and the next steps

**Fig. 3:**
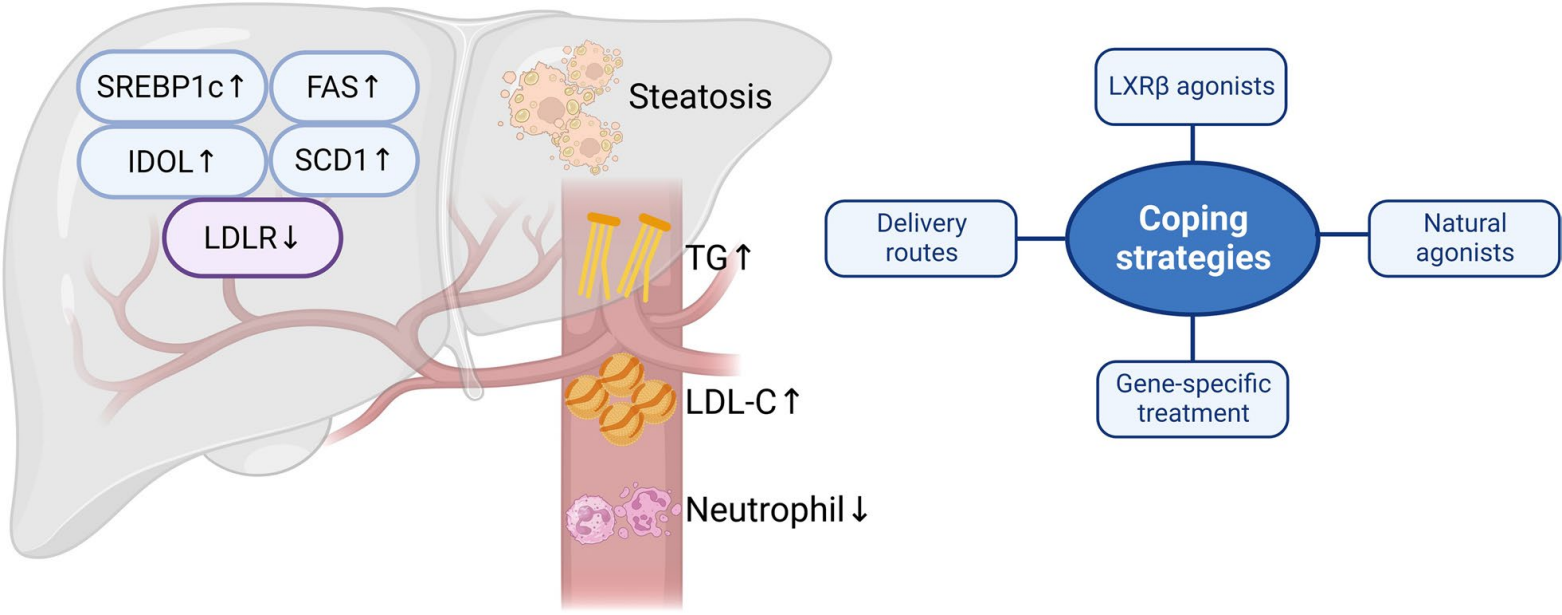
Potential adverse effects of LXR agonists and next steps. Systemic administration of LXR agonists may cause neutropenia and lipogenesis related adverse effects such as hyperlipidemia and hepatic steatosis. Investigations in regional drug delivery, LXRβ selective agonists, natural ligands and LXR downstream gene specific treatment may help alleviate the adverse effects and promote the clinical translation. *FAS* fatty acid synthase, *IDOL* inducible degrader of low-density lipoprotein receptor, *SCD1* stearoyl-CoA 9-desaturase, *SREBP1c* sterol regulatory element-binding protein 1c

Although LXR agonists are very promising therapeutics from the results of preclinical research, their adverse effects have hindered their clinical translation (Fig. 3). Oral administration of LXR agonist GW3965 or T0901317 elevated serum level of triglyceride and low-density lipoprotein cholesterol in mice and hamsters, which are attributed to LXR-induced elevation of lipogenesis related genes in liver, including sterol regulatory element-binding protein 1c (SREBP1c), Fatty acid synthase (FAS) and Stearoyl-CoA 9-desaturase (SCD1) [5]. Moreover, LXR activation can also upregulate IDOL, which may contribute to the reduction of low-density lipoprotein receptor in liver, leading to increased LDL level [16]. All these mechanisms likely contribute to dyslipidemia and hepatic steatosis in humans on LXR agonists during clinical trials. To overcome this, the use of LXRβ selective agonists has been proposed, as the liver related adverse effects of LXRs seem to be mediated mainly by LXRα in mice [80]. BMS-852927, a LXRβ-selective partial agonist with 20% LXRα and 88% LXRβ activity compared to the full pan agonist T0901317 did not change the level of TG and LDL-C at doses with therapeutic effects after 14-day treatment in cynomolgus monkeys and mice [81]. However, the lipogenesis effects remained including significant increased plasma triglyceride and low-density lipoprotein—cholesterol when reverse cholesterol transport associated benefits were observed in human [81]. Moreover, a drop of circulating neutrophil count was observed in volunteers treated with BMS-852927 [81], which is consistent with the data from preclinical studies [48]. In addition, another clinical trial using single ascending dose of a different agonist LXR-623 was stopped due to CNS related adverse effects, while beneficial effects such as elevated plasma ABCA1 and ABCG1 were also reported [82].

However, the clinical trials mentioned here both systemically administrated the LXR agonists by oral delivery since they were testing the potential and safety to treat atherosclerosis related conditions [81, 82], which probably caused more undesirable effects in liver and hematopoietic system. For CNS diseases, it is feasible for regional delivery so that the systemic adverse effects can be diminished. A nasal delivery of a synthetic LXR agonist N,N-dimethyl-3β-hydroxycholenamide (DMHCA) alleviated cognitive impairment and increased brain ApoE content without changing the serum level of cholesterol and triglycerides in the Alzheimer’s disease model of McGill-Thy1-APP transgenic mice [24]. And in situ delivery seems viable for cerebrovascular diseases accompanied with surgical procedures like stent implantation or hematoma removal. Moreover, compared to non-sterol synthetic agonists such as T0901317 and GW3965, steroidal agonist DMHCA seems to activate SREBP1c less while upregulating ABC transporters [24], which may suggest it is possible to design gene specific LXR agonists to cope with different pathologies. In addition, some natural LXR agonists and oxysterol precursors like squalene deserve attention as they may have less unfavourable effects [74, 83]. Dietary Sargassum fusiforme-derived lipid extract was found to be a potent LXRβ agonist, which improved short-term memory and reduced hippocampal β-amyloid plaque of AD mice [84]. Some small molecules were found to upregulate LXR downstream genes including ABCA1 and ApoE without direct LXR agonism [85], which is worth more investigation for targeted therapies without adverse effects.

## Concluding remarks

**Fig. 4:**
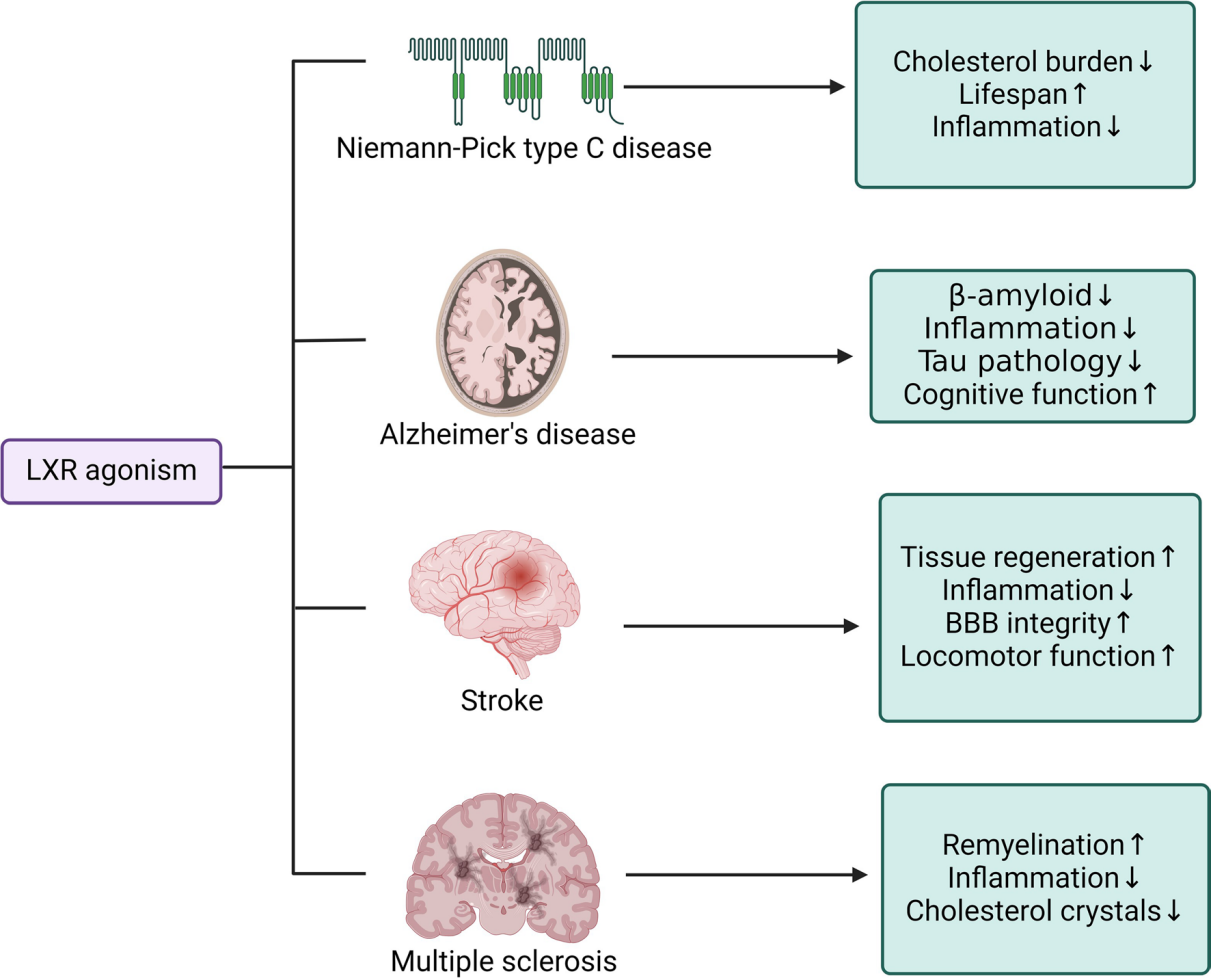
Potential benefits of LXR agonism from preclinical studies in different CNS pathologies

In summary, the activation of LXR signaling has the potential to help with various neurological disorders including genetic, immune-mediated, neurodegenerative and vascular pathologies by modulating lipid metabolism, cholesterol recycling, inflammatory response and integrity of the blood–brain barrier (Fig. 4). LXR agonism inhibits the expression of proinflammatory genes to reduce tissue damage and provide a repair-favorable environment; while enhanced phagocyte cholesterol efflux and cholesterol recycling offer material for regeneration, and reduced cholesterol/lipid accumulation also alleviate inflammation mediated injury. Moreover, LXR activation may protect cellular membrane from oxidative stress injury in CNS pathologies by improving membrane components, which deserves more investigations. However, challenges of affecting LXR remain due to systemic side effects caused by globally distributed LXR-expressing cells. Hopefully in the future, new compounds or methods of LXR-signaling regulation with higher CNS- and transcriptional selection can be developed to facilitate clinical translation of LXR signaling in neurology.

## Data Availability

Not applicable.

## Glossary

Alzheimer’s disease (AD): a progressive neurodegenerative disease; the most common cause of dementia.
AP-1: a transcription factor, regulates inflammatory response.
Apolipoprotein E (ApoE): a major cholesterol carrier that supports lipid transport and injury repair in the brain; the ε4 allele is a risk factor of Alzheimer’s disease.
ATP-binding cassette transporter sub-family A and G (ABCA, ABCG): the major transporters regulating cellular cholesterol and phospholipid homeostasis; transcriptionally regulated by LXRs.
Blood–brain barrier (BBB): a sealed barrier isolating the brain from the rest of the body that maintains brain homeostasis by regulating the entry of molecules into the brain, composed of endothelial cells, pericytes, capillary basement membrane, and astrocyte end-feet.
Central nervous system (CNS): the brain and spinal cord.
COX-2: the enzyme catalyzes the synthesis of prostanoids, a hallmark of inflammatory response in tissue.
Experimental autoimmune encephalomyelitis (EAE): the most commonly used experimental animal model for the human inflammatory demyelinating disease, multiple sclerosis.
IL-1β: a potent proinflammatory cytokine mediating pyroptosis.
Inducible degrader of LDLR (IDOL): an E3-ubiquitin ligase that causes ubiquitination and degradation of LDLR in lysosome and is a regulator of LDLR expression.
iNOS: an enzyme generating nitric oxide from the amino acid L-arginine; a hall mark of proinflammatory microglia/macrophages, can exacerbate oxidative injury during pathological neuroinflammation.
Intracerebral hemorrhage (ICH): a type of stroke caused by the burst of arteries within the brain parenchyma followed by devastating outcome.
Liver X receptor (LXR): orphan nuclear receptor with two isotypes α and β, the key regulator of lipid metabolism and inflammatory response.
Low-density lipoprotein receptor (LDLR): a cell-surface receptor that mediates the endocytosis of cholesterol-rich low-density lipoprotein.
Monocyte chemoattractant protein-1 (MCP-1/CCL2): one of the key chemokines that regulate migration and infiltration of monocytes/macrophages.
Multiple sclerosis: a chronic progressive autoimmune demyelinating disease, in which the CNS myelin sheath and axons are damaged, resulting in physical, cognitive and psychiatric symptoms.
NFκB: a key transcription factor required for a large number of inflammatory genes; related to proinflammatory activation of microglia/macrophages.
NOD-, LRR- and pyrin domain-containing protein 3 (NLRP3): an intracellular sensor that detects a broad range of microbial motifs, endogenous danger signals and environmental irritants, resulting in the formation and activation of the NLRP3 inflammasome.
STAT1: a transcription factor that can induce proinflammatory-gene expression and drive the proinflammatory phenotype of microglia/macrophages in CNS injury.
Stroke: an acute cerebrovascular event caused by disruption of blood supply to the brain, resulting in lasting brain damage, long-term disability, or even death.
SUMOylation: a process in which SUMO proteins are covalently attached to specific lysine residues in target proteins, thereby regulating various aspects of protein function, including transcription, subcellular localization, DNA repair and cell cycle.
TNFα: a powerful pro-inflammatory agent that regulates many facets of microglia/macrophage function.
Triggering receptor expressed on myeloid cells 2 (TREM2): a membrane protein that regulates key myeloid cell functions including phagocytosis and chemotaxis.
β-amyloid (Aβ): the main component of the amyloid plaques found in the brains of people with Alzheimer's disease.
